# Case Report: ST-Elevation Myocardial Infarction in Third Trimester Pregnancy

**DOI:** 10.5811/cpcem.41487

**Published:** 2025-05-01

**Authors:** Luis Martinez, Emmelyn J. Samones, Michael Kiemeney, William Michael Downes

**Affiliations:** Loma Linda University Medical Center, Department of Emergency Medicine, Loma Linda, California

**Keywords:** acute myocardial infarction, spontaneous coronary artery dissection, ST-elevation myocardial infarction, pregnancy, case report

## Abstract

**Introduction:**

While rare in pregnancy, acute coronary syndrome (ACS) does happen. It has been found to be more common in individuals with risk factors. A case of chest pain in a previously healthy female in her third trimester demonstrates the importance of keeping ACS high on the differential list.

**Case Report:**

A 26-year-old pregnant female gravida five, para three at 37 weeks gestation with a past medical history of diet-controlled gestational diabetes, obesity, and family history of myocardial infarction (MI) presented to an outside hospital for chest pain and was transferred to the closest ST-elevation myocardial infarction (STEMI) receiving emergency department (ED) after she was found to have an electrocardiogram (ECG) concerning for acute STEMI. On arrival to the ED, STEMI protocol was activated based on ST-segment elevations on inferior and antero-lateral leads on the ECG. Bedside assessment of the fetus by obstetrics showed a viable intrauterine pregnancy, and the patient was taken to the cardiac catheterization lab. She was found to have a 100% thrombotic occlusion in the ostium of the right posterolateral artery, and percutaneous coronary intervention was performed. The patient was discharged with plans for cesarean section at 39 weeks.

**Conclusion:**

This case highlights the need for early STEMI activation and consultation with obstetrics when a pregnant patient presents with an ECG suggestive of STEMI. It also emphasizes the importance of maintaining a high level of suspicion for STEMI in pregnant patients presenting with chest pain. Although rare—0.6 in 10,000 pregnancies—mortality rates range from 5.1–37% throughout pregnancy and postpartum. It is important to remember that pregnancy does not preclude a patient from undergoing standard treatment of acute MI.

## INTRODUCTION

Chest pain is a common chief complaint that emergency physicians should be comfortable addressing and risk-stratifying. While the overall risk of myocardial infarction (MI) in the general childbearing-age population is low, a complaint of “chest pain” should heighten the senses of emergency physicians.^2^ On the other hand, acute MI during pregnancy is more common when compared to a similar non-pregnant population.^3^ Additionally, one should keep in mind that spontaneous coronary artery dissection (SCAD) has been reported as one of the most common causes of acute coronary syndrome (ACS) in pregnant patients.^3^,^4^ However, the risk factors that are commonly associated with acute ST-elevation myocardial infarction (STEMI) during pregnancy differ from those associated with SCAD.^5^,^6^

From an epidemiological standpoint it is important to maintain a frame of reference but always keep in mind atypical presentations. There are, however, common risk factors that predispose women to acute MI during different stages of pregnancy. Atherosclerosis plays a bigger role in first trimester MI, atherosclerotic and thrombosis for second trimester, and SCAD during third trimester with SCAD being the most common cause at any point during pregnancy.^5^,^7^,^8^ As is evidenced by our case report, the patient described falls outside the previously reported pathophysiological causes of MI in the third trimester.

## CASE REPORT

**Image 1:**
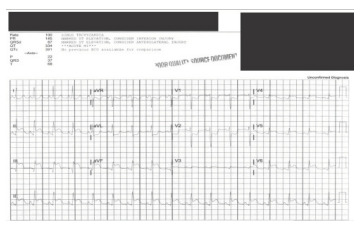
Electrocardiogram demonstrating ST-elevation myocardial infarction (STEMI). Leads II, III, aVF, V3–V6 demonstrate marked ST-segment elevation consistent with STEMI, and leads aVL, V1–V2 show marked ST-segment depression consistent with reciprocal ECG changes.

A 26-year-old female gravida five, para three at 37 weeks gestation with a past medical history of diet-controlled gestational diabetes, obesity, and a sister who had died from an MI at age 30 presented to an outside hospital labor and delivery, where she reported acute onset of mid-chest pain while grocery shopping. The patient was transferred to a STEMI-receiving ED due to concerns for STEMI on the initial electrocardiogram (ECG) (Image 1). Prior to arrival, the patient received aspirin and morphine at the outside hospital.

**CPC-EM Capsule**

What do we already know about this clinical entity?

*Acute myocardial infarction (MI) is a rare condition in pregnancy, typically caused by spontaneous coronary artery dissection (SCAD) in the third trimester*.

What makes this presentation of disease reportable?

*This case report highlights a pregnant patient in her third trimester who presents with acute MI secondary to thrombosis and atherosclerosis, conditions typically seen in the first and second trimester*.

What is the major learning point?

*Acute MI can occur during the third trimester of pregnancy and is not always attributed to SCAD*.

How might this improve emergency medicine practice?

*It is important to maintain a high level of suspicion for MI in pregnant patients presenting with chest pain*.

**Image 2:**
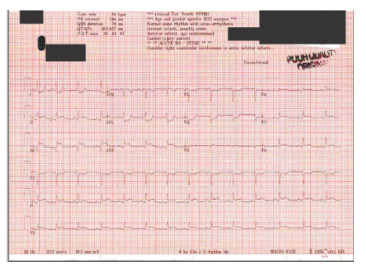
Electrocardiogram obtained at time of patient’s arrival to cardiac receiving center. Leads II, III, aVF show similar ST-segment elevation compared to initial ECG. Leads I and aVF show progression in ST-segment depression. V1 shows improved ST-segment depression but similar V2 depression. There is improved ST-segment elevation in Leads V3–V6.

Vitals on arrival were blood pressure 131/83 millimeters of mercury, heart rate 99 beats per minute, respiratory rate 25 breaths per minute, and oxygen saturation 99% on room air. The ECG in the ED showed ST elevation in leads II, III, V3, V6, and aVF with reciprocal depressions in V1, V2, I and aVL (Image 2). Laboratory results revealed elevated troponin at 0.07 nanograms per milliliter (ng/mL) and 6.04 (ng/mL) (reference range: ≤0.03 ng/mL) and normal glucose of 114 milligrams per deciliter (mg/dL) (70–140 mg/dL). Point-of-care ultrasound performed by the cardiology team demonstrated a hypokinetic inferior wall. Fetal assessment showed fetal heart rate of 130 beats per minute (110–160 beats per minute). Ultimately, the patient was emergently taken to the cardiac catheterization lab. She was given clopidogrel 600 milligrams (mg) and 10,000 units of heparin total (3,000 intra-arterial followed by 7,000 units intravenous).

The cardiac catheterization lab report showed 100% thrombotic occlusion in the ostium of the right posterolateral artery (rPL) without other evidence of coronary artery disease. Percutaneous coronary intervention (PCI) was performed to the rPL vessel with a 3.0 x 18 millimeter drug-eluting stent (DES). No coronary artery dissection was evident. During hospitialization, the patient was followed by obstetrics (OB) who initially recommended labor induction at 39 weeks. Obstetrics later recommended elective cesarean section due to suspected fetal macrosomia, gestational diabetes mellitus, and obesity with high risk for shoulder dystocia. The patient did not experience complications and was discharged home from the cardiology service with instructions to continue clopidogrel 75 mg daily, aspirin 81 mg daily, and metoprolol 12.5 mg daily.

Prior to the scheduled C-section, a transthoracic echocardiogram was obtained, which found “mild hypokinesis and preserved thickness of the basal inferior (posterior) wall and mild hypokinesis and preserved thickness of the mid inferior wall, with an ejection fraction of 65%.” The patient was later admitted to the OB service; she underwent a C-section under general anesthesia, and a healthy nine-pound, 7.3-ounce male was delivered. There were 700 mL estimated blood loss reported without major complications. On day seven, after an uneventful postoperative course, the patient was discharged home.

## DISCUSSION

Acute MI is a rare condition in pregnancy. However, reports indicate that MIs during the third trimester are often associated with SCAD. Acute MI in a pregnant patient is statistically more common in multigravidas, with a prevalence of 66%, but it is particularly higher in patients who are >30 years of age, with incidence of 72%. Additionally, these cases often involve the anterior wall, accounting for 78% of occurrences.^7^ The history of gestational diabetes, obesity, and family history of MI put this patient at a higher risk of ACS.

Given that SCAD is most common in the third trimester of pregnancy, it is important to risk-stratify patients whose presentations are concerning for acute MI in the peripartum or postpartum period. Also, without an angiogram, SCAD can only be suspected based on previously described risk factors; for that reason, closed loop communication with cardiology is necessary to guide acute phase treatment. Regarding medication treatment options, there are several working theories. One theory postulates that bleeding from the vasa vasorum creates an intramural hematoma in the coronary arteries leading to myocardial ischemia.^9^ For that reason, if SCAD is suspected or confirmed, the continued use of anticoagulation and antiplatelet therapy should be avoided unless there is confirmation of thrombus or there are other systemic indications.^10^ If anticoagulation is continued, there is a theoretical risk of worsening intramural hematoma and extension of the dissection.^10^ Another theory argues that there is likelihood from an anatomical standpoint, as evidenced by the lack of inflammatory cells in tissue studies, that SCAD might be precipitated by impairment of endothelial repair, which is exacerbated during pregnancy due to low levels of estrogen.^9^ It fully advocates for anticoagulation and antiplatelet treatment if no other contraindications exist.^9^ This stands in contrast to the mechanical forces theory underlying the intramural hematoma theory.

Ultimately, if diagnostic studies such as coronary angiogram reveal thrombotic occlusion causing MI, then PCI and aggressive medical management are indicated as described in this case report. Glycoprotein blockers have higher binding efficacy to abciximab over eptifibatide and tirofiban, particularly during lactation, and most thrombolytics seem to be compatible with pregnancy; however, human data is still limited.^8^

## CONCLUSION

The leading cause of STEMI and non-STEMI during the peripartum and postpartum period in the third trimester is spontaneous coronary artery dissection. As in the general population other causes such as advancing maternal age and history of atherosclerosis and thrombosis are bigger risk factors during the first and second trimesters. While the pathophysiology of SCAD has not been fully elucidated, a low estrogen state seems to play a major role in the impairment of endothelial repair leading to plaque formation and thrombotic events. Interventions such as computed tomography of the coronaries, coronary angiography, and percutaneous coronary intervention should not be withheld if indicated.

Medical management including aspirin, beta blockers, anticoagulation, selective antiplatelets, and thrombolytics if indicated should not be withheld to treat an acute MI in the peripartum or partum period. Finally, the early consultation of OB and cardiology is important if acute coronary syndrome is suspected. If the patient is pregnant and in her third trimester, emergency physicians should discuss the increased likelihood of SCAD. This does not change the initial management in the acute phase of treatment. If SCAD is noted on coronary angiography, cardiology will then decide on optimal medical treatment vs PCI. If interventional cardiology is not available at the time of STEMI diagnosis, the patient should be promptly transferred to a STEMI center preferably with in-person or telephone OB consult services available.
